# Nude and Modified Electrospun Nanofibers, Application to Air Purification

**DOI:** 10.3390/nano13030593

**Published:** 2023-02-01

**Authors:** Patricio J. Espinoza-Montero, Marjorie Montero-Jiménez, Stalin Rojas-Quishpe, Christian David Alcívar León, Jorge Heredia-Moya, Alfredo Rosero-Chanalata, Carlos Orbea-Hinojosa, José Luis Piñeiros

**Affiliations:** 1 Escuela de Ciencia Químicas, Pontificia Universidad Católica del Ecuador, Quito 17012184, Ecuador; 2 Facultad de Ciencias Químicas, Universidad Central del Ecuador, Quito 170521, Ecuador; 3 Centro de Investigación Biomédica (CENBIO), Facultad de Ciencias de la Salud Eugenio Espejo, Universidad UTE, Quito 170527, Ecuador; 4 Departamento de Ciencias Exactas, Universidad de Las Fuerzas Armadas ESPE, Av. Gral. Rumiñahui S/N, Sangolquí P.O. Box 171-5-231B, Ecuador

**Keywords:** electrospinning, air purification, filtration, nanofibers

## Abstract

Air transports several pollutants, including particulate matter (PM), which can produce cardiovascular and respiratory diseases. Thus, it is a challenge to control pollutant emissions before releasing them to the environment. Until now, filtration has been the most efficient processes for removing PM. Therefore, the electrospinning procedure has been applied to obtain membranes with a high filtration efficiency and low pressure drop. This review addressed the synthesis of polymers that are used for fabricating high-performance membranes by electrospinning to remove air pollutants. Then, the most influential parameters to produce electrospun membranes are indicated. The main results show that electrospun membranes are an excellent alternative to having air filters due to the versatility of the process, the capacity for controlling the fiber diameter, porosity, high filtration efficiency and low-pressure drop.

## 1. Introduction

Air pollution is a global problem that has gained special attention recently, mainly because it is tough to control once a pollutant is released into the atmosphere [1]. Particulate matter (PM) is one of the air pollutants of primary concern, it is a complex mixture of airborne particles (solid, liquid, organic, and inorganic) that can transport different disease-causing microorganisms [2], for instance: Saliva microparticles, dust, pollen, vehicle emissions gases, viruses, and bacteria, etc. [3]. These particles have been divided depending on their diameter, so PM_0.3_, PM_2.5_, and PM_10_ indicate that the diameter of the particles is less than 0.3, 2.5, and 10 μm, respectively [4,5,6]. Some researchers have studied the adverse effects of PM on human health, and [7,8] reported that PM is closely related to the development of cardiovascular and respiratory diseases. Thus, most research on air purification primarily focuses on the source of pollution to prevent the spread of pollutants [2,3].

Air purification technologies are generally based on ozonation, photocatalytic oxidation, ionizers, adsorption, electrostatic precipitators, and solid medium filtration [9], with filtration being the most efficient process for removing PM from the air [10]. However, conventional filters are composed of synthetic materials such as fiberglass, polyester, and polypropylene [9], which are not capable of retaining the finest particulate matter that is dangerous for humans’ health [11].

The development of nanomaterials can retain the minor PM with greater efficiency. Nanofiber-based membranes are suitable for this since having thinner fibers mean an increase in area per unit mass. The resulting membranes are of very low weight, high filtration efficiency and low pressure drop. In this context, electrospinning has gained popularity because of its novelty procedure to form fibrous, porous and very thin membranes. Using this technique, it is possible to obtain fibers with a diameter from 10 to 100 times smaller than those produced by melting yarn [12,13]. Membranes produced by electrospinning generally have the following characteristics: (i) High porosity due to interconnected structure, (ii) improved specific surface area, (iii) unique topography, and (iv) different surface chemical properties that depend on the chemical composition [14].

Furthermore, the electrospinning equipment consists of a syringe and a collector, between which an electric field is applied. The syringe contains polymer in a liquid state or in solution, which is executed in a controlled manner thanks to the effect of the high-voltage electric field. This assumes the configuration of the “Talyor’s cone” and lengthens because the force of the electric field exceeds the electrostatic forces [15,16]. During this process, the fluid is electrostatically charged, takes the form of a jet, is attracted by the collector where the fibers accumulate, and the solvent evaporates since it leaves the syringe until it reaches the collector [17].

In addition, this technique is very versatile because a variety of modifications can be applied, so that the fiber can fulfill a specific function, for example, nanofibers can be functionalized by binding active species such as nanoparticles, different fiber diameters can be achieved by manipulating the processing parameters, and they can also be assembled on a variety of matrices by using their alignment, stacking or folding. In this way, composite membranes and multi-level structured membranes can be produced even with antibacterial properties and good thermal stability [2,3,18]. Additionally, membranes with beads can be produced; the beads are protrusions formed on the fibers because of a rupture of the jet either by working with high flow rates or high voltages. The presence of beads can contribute to reduce the pressure drop due to changes in membrane packing density. Nevertheless, not much is known about the effect of dots on filtration efficiency, which could be a disadvantage [19].

Additionally, the wettability/hydrophilicity of membranes depends on the polymer and solvent selected for the preparation of polymeric solution, so both have to be carefully chosen [20]. The polymer should not have high viscosity because it will produce a plugging in the needle, so in the case of using polymeric solutions, it is recommended to use a volatile solvent because it can evaporate [21]. Moreover, other parameters that have a strong influence in membrane production are: (i) Voltage applied, (ii) distance between the needle and collector, (iii) type of collector, and (iv) the diameter of the needle [21]. In addition, surface tension, Coulomb repulsion force, electrostatic and viscoelastic forces, gravity, and air drag influence the morphology and porosity of thinner nanofibers especially in [15,22].

For that reason, this technique can be modified according to the membranes requirements, for example, to avoid problems of plugging and low productivity the electrospinning system can be used without needle, but this system requires the application of a greater electric field [18,23]. Another configuration is the use of multi-needled electrospinning with the purpose of controlling the distribution of fibers and increasing the speed of production, however the disadvantage of this system is the possibility of plugging the needles and the interaction between the jets of each needle [24]. Additionally, for developing electrospinning membranes, it is possible to work with different polymer matrices, which include natural and synthetic polymers. The most common natural materials are chitosan cellulose and starch [25], while the most outstanding synthetic polymers are polyacrylonitrile (PAN), polysulfone (PSU), polyvinyl chloride/polyurethane (PVC/PU) among others [26].

**Figure 1:**
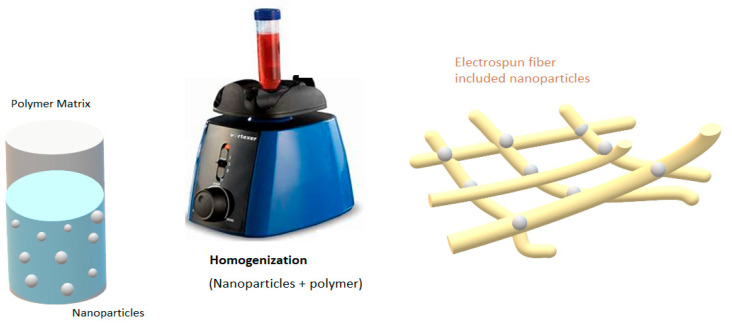
Nanoparticles included into the polymer matrix for the obtained electrospun.

Finally, the membranes obtained by electrospinning have been studied in several applications. Among them are mainly applications in water purification such as microfiltration, ultra-filtration, nanofiltration, and osmotically driven membrane, as well as the membrane bioreactor, membrane distillation, aqueous/oil phase separation, heavy metal adsorption and antimicrobial processes [16,22]. These last ones are very interesting membranes because its antimicrobial properties are given by the introduction of nanoparticles, which have this antimicrobial property, e.g., Ag, Zn, Ce, etc. The nanoparticles can be integrated using two methodologies; these can be included into the polymer matrix (Figure 1) or these can be impregnated by adsorption after the manufacture of the fiber or before it. In the first case, the nanoparticles and the polymer must form a suspension, which would be used for the formation of the fiber [27,28].

Additionally, it is important to highlight three review papers that have been previously published in this area. Namely, Lu et al., 2021, provides information on the main operating conditions for obtaining electrospun fibers. In particular, cellulose fibers, Zeina, PAN, PV, PC and Nylon are mentioned [29]. However, Kadam et al., 2018, reports the processes and operating conditions for obtaining fiber, in addition to the characterization and morphology of the fiber, emphasizing the use of PLA, PA and PAN [30]. Finally, Mamun et al., 2021, focuses their review on retention mechanisms for air filtration and the use of materials of natural origin to obtain multifunctional bio-based nanofibers [31].

Based on the above, this review addresses a compilation of electrospinning studies focused on synthesizing polymers and membranes to be applied in air filtration. This revision summarizes the kind of polymers used to electrospinning applications and its production, otherwise it is addressed the study of several modifications to synthesize fibers and membranes and different changes in experimental parameters such as the polymer solution or in the technique used in electrospinning equipment. Finally, the electrospun membrane applications are reported.

## 2. Nude Electrospun Nanofibers

### 2.1. Synthesis of Polymers for Electrospun Nanofiber Membranes

**Figure 2:**
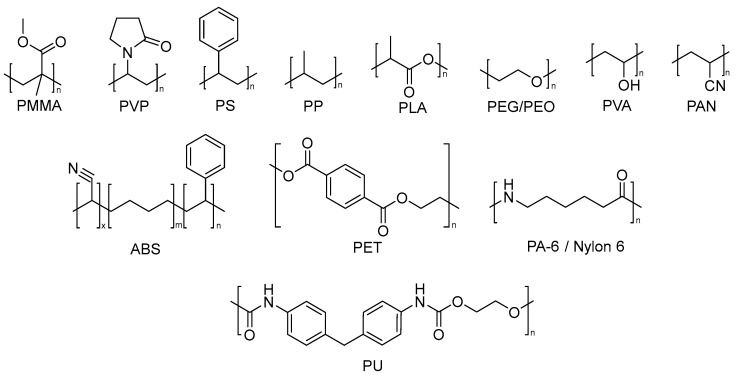
Molecular structure of the main polymer structures used for the development of electrospun nanofibers.

The use of nanofiber a filtering medium is well established, and the electrospun nanofiber have several applications such as electrospun fibers for air purification [32,33,34] and air filtration media [35]. The different characteristics of nanofibers as morphologies, mechanical and optical properties, thermal stability, electrical conductivity, photocatalytic activity and bioactivity underlie their macromolecular structure and chemical composition [36]. The most commonly used polymers for the development of electrospun nanofiber membranes are shown in Figure 2.

#### 2.1.1. Polymethyl Methacrylate (PMMA)

**Figure 3:**
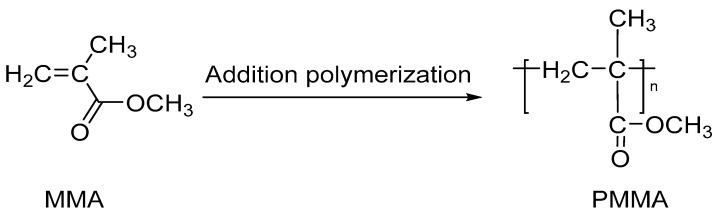
Synthesis of PMMA.

The PMMA is a transparent thermoplastic polymer, obtained from methyl methacrylate (MMA) and an ester of poly(methacrylic acid) [37] (Figure 3). This polymer is usually synthesized by the radical polymerization of MMA, and anionic initiations, polymerization by the addition and atoms transfer radical polymerization (ATRP) are also available [38,39]. The polymeric chain contains hydrophobic (methylene) and hydrophilic (carbonyl) groups in each monomeric unit [39]. This polymer could be modified to enhance the capture of VOCs [40] using cyclodextrins (as α-cyclodextrin (α-CD), β-cyclodextrin (β-CD) and γ-cyclodextrin (γ-CD)) due the ability to form host-guest inclusion complexes). In addition, the PMMA shows potential as an encapsulation material due its high chemical stability, biocompatibility and nontoxicity [41].

#### 2.1.2. PVP Polyvinylpyrrolidone

**Figure 4:**
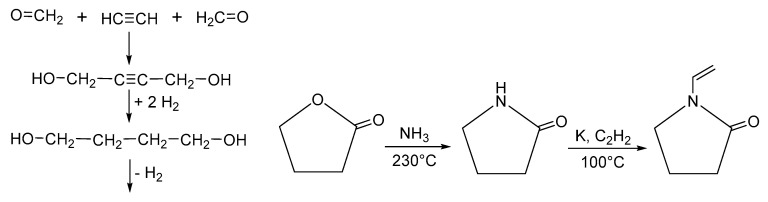
Synthesis of N-vinylpirrolidone.

Polyvinylpyrrolidone (PVP), polyvidone or povidone is a water-soluble polymer synthesized from monomers of N-vinylpyrrolidone [42]. The Reepe’s reaction is a convenient process that allows us to obtain soluble polyvinylpyrrolidone. The synthesis strategy initially uses acetylene and formaldehyde to obtain 1,4-butine diol, which is later hydrogenated to butane diol. Butyrolactone is obtained by oxidative cyclization and the subsequent reaction with ammonia afford, pyrrolidone. Finally, the vinyl group is introduced using acetylene to form N-vinyl-2-pyrrolidone-2 (Figure 4).

The radical polymerization in water or 2-propanol, using hydrogen peroxide, AIBN or organic peroxide as initiators, is the main mechanism of synthesis. Give that the molecular weight of PVP is regulated by the concentration of hydrogen peroxide, the concentration of alkali hydroxide and copolymerization processes can be obtained with a lower molecular weight, insoluble polyvinylpyrrolidone (Crospovidone) and vinylpyrrolidone-vinyl acetate copolymer (Copovidone), respectively [43,44].

PVP is a versatile polymer with interesting and convenient features, it has an excellent solubility in solvents of different polarities, and it is used to stabilize suspensions and emulsions. PVP is a polymer recognized as safe by the Food and Drug Administration (FDA), it is a biocompatible and non-toxic polymer widely used in food industry, medicine, pharmaceutical and biomedical applications [44,45,46]. In particular, PVP showed effective particulate matter (PM_2.5_) capture in a transparent air filter, and development was by the electrospinning method. The study of PM capture evidences a correlation between the type of polymer and the dipole moment where PVP (2.3 D) and PAN (3.6 D) have a better capture of PM 2.5 [47].

#### 2.1.3. Polyacrylenitrile (PAN)

**Figure 5:**
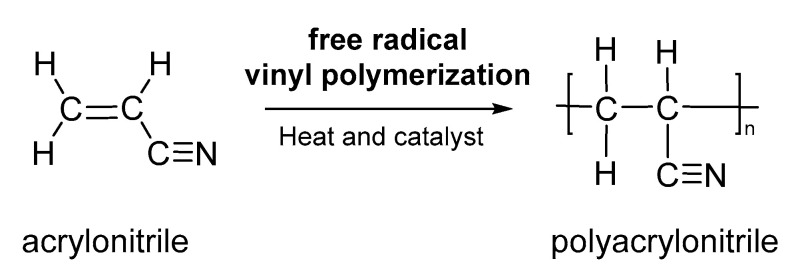
Polymerization of acrylonitrile (AC) to PAN.

PAN and copolymers of PAN are polymers with versatile and multipurpose applications. Due to its high carbon yield (up to 56%), PAN is used as a carbon fiber precursor, and taking into account that it has interesting chemical, mechanical and thermal properties that allow for electrospun nanofibers and prepare carbon nanostructure microspheres. Nanocomposite fibers and polyacrylonitrile blends can be used in the manufacture of protective materials, technical textile, air filtration material, antimicrobial nanofibers spun for water treatment, electrochemical sensors, drug delivery, among others [48,49]. PAN can be synthesized by free radical vinyl polymerization (Figure 5), heat or by a catalyst from acrylonitrile monomer and was marketed by DuPond as-spun fiber around 1941 [48]. This polymer can also be synthesized by atom transfer radical polymerization, radicals catalyst and anionic polymerization with butyllithium that yield atactic polymers [49,50,51,52]. Due to its high carbon yield (up to 56%), PAN is used as a carbon fibre precursor, nanoparticles by the dispersion/emulsion polymerization process, electrospun nanofibers and carbon nanostructure microspheres with several applications such as antimicrobial nanofibers spun for water treatment, electrochemical sensors, supercapacitors materials, drug adsorption and air filtration media [52,53,54,55,56,57].

#### 2.1.4. Polystyrene (PS)

**Figure 6:**
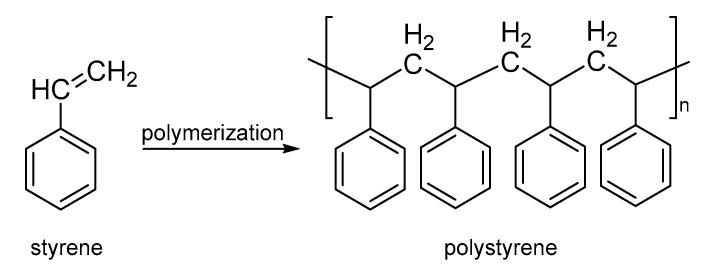
Synthesis of PS.

PS is an aromatic hydrocarbon polymer that results from the addition of polymerization of the styrene monomer (Figure 6) [58]. Modern PS production began in the 1970s due to the continued utility of the product and several applications ranging from food and medical packaging to home insulation [59]. Structural properties such as tacticity, molecular weight, mechanical strength, water and oxygen barrier, dimensional stability and thermal stability have attracted great interest from academicians and industrialists. Nevertheless, the disposal of these products creates environmental pollution because of their nondegradable nature. In this sense, a series of reviews and new technologies propose their recycling and reuse by mechanical (new nanocomposites), chemical (photodegradation and photostabilization) and thermal recycling (pyrolysis) [60,61,62,63]. Particularly, PS allow to build by electrospinning process fibers and nanofibers from polymer solution. The effect of PS concentration, applied voltage and spinning distance generate several morphologies of fibers with convenient mechanical properties [64,65,66]. Some of the applications based on PS include the nanofiber membrane with superhydrophobicity and superoleophilicity for the selective separation of water and low viscous oil [67], functionalization with acrylamide using plasma by development electrospun PS nanofibers [68], electrospun PS nanofibers as novel adsorbent to transfer an organic phase from an aqueous phase [69,70], grooved PS fibers by electrospinning and their effect in solvents [71] and PS nanofibers applied in the filter media [72].

#### 2.1.5. Polyvinyl Alcohol (PVA)

**Figure 7:**
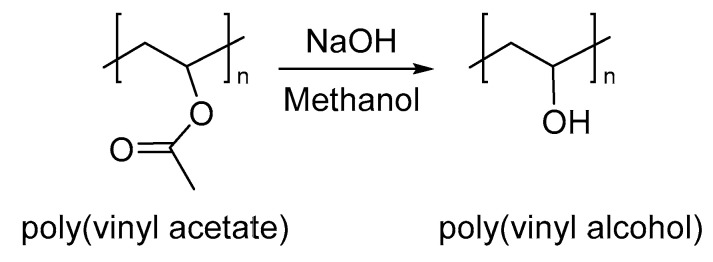
Synthesis of PVA.

PVA is a water-soluble synthetic polymer, prepared by the hydrolysis of polyvinyl acetate in ethanol with hydroxide (Figure 7) [73], unlike most vinyl polymers, where the polymerization is developed by the corresponding monomer (vinyl alcohol). PVA is a biocompatible polymer used in biodegradable packing for food preservation, skin care applications and potential biomedical applications [74,75]. However, the tendency to absorb moisture limits its use under high moisture conditions. In this sense, several studies seek to improve its properties development blends with other polymers and additives such as citric acid, succinic acid, and tartaric acid [76,77].

#### 2.1.6. Polypropylene (PP)

**Figure 8:**
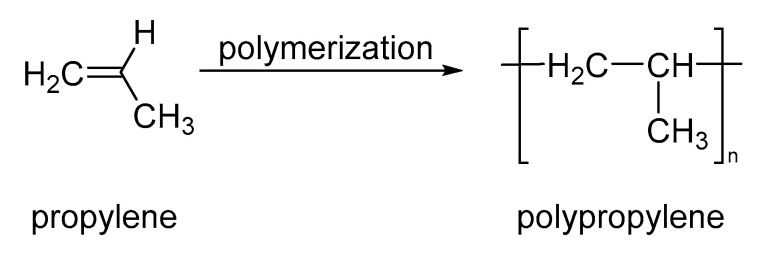
Synthesis of polypropylene.

The synthesis of PP from propene by chain-growth polymerization (Figure 8) has been known since the 1950s and was initially studied by Hogan and Banks. The potential applications of PP show a dependence on mechanical properties and thermal resistance that were improved by relevant advances of Natta and Ziegler, who developed a stereospecific polymerization using organometallic catalysts arising isotactic PP [78]. Considering that PP is a cheap polymer with excellent processability, chemical resistance, and moisture barriers, the chemical development favored the global industrial production of PP, which was 50 Mt in 1976 and grew to 360 Mt in 2018 [79]. However, the synthesis and functionalization of PP is an interesting current study area, where the obtention of novel polymers with different tacticity allow for potential applications in textile, automotive, cosmetics, and consumer packaging. In this sense, the polydispersity and molecular weight are controlled using transition metal (N,N-dietil hafnio) and metal alkyl chain transfer agent as ZnEt_2_ to produce amorphous atactic polypropylene (a-PP) with narrow polydispersity and molecular weights of 12.6 kDa to 111 kDa [78]. Moreover, PP shows interesting and convenient mechanical properties as polyethylene–polypropylene blends and polypropylene nanocomposite fibers [79,80].

#### 2.1.7. Polylactic Acid (PLA)

**Figure 9:**
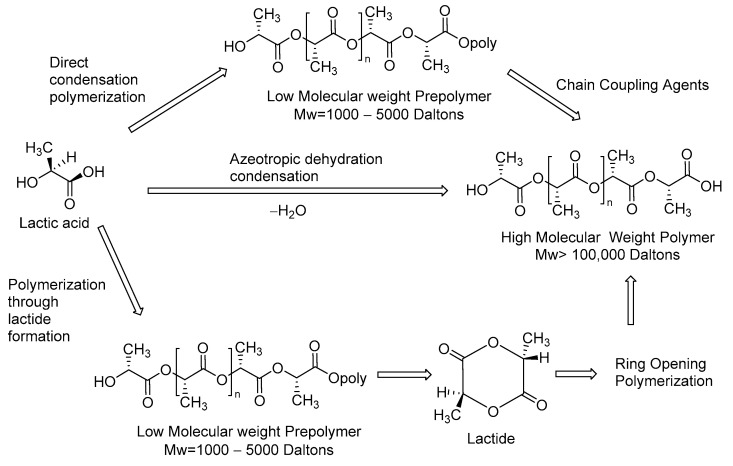
Synthesis of polylactic acid.

Lactic acid is the building block for the polymerization of PLA. The synthesis can be performed by different methods as direct condensation polymerization and polymerization through lactide formation and azeotropic dehydration condensation (Figure 9) [81,82]. The enantiomers of lactic acid (L-(+)-LA and D-(-)-LA) are employed indistinctly in industrial production, and the polymer obtained is classified as an aliphatic polyester due to ester bonds that connect the monomer units. This structural characteristic plays a key role in non-toxic degradation process, applications in biomedical field and development of renewable and biodegradable materials [83]. However, the convenient mechanical and thermal properties allow for the development of nanofibers prepared by electrospinning that have morphological and structural features for biological applications like a scaffolds for tissue engineering, nanofibers of thin films of PLA/paclitaxel as molecular carriers for the sustained release of cancer therapeutics and electrospun PLA-cyclodextrins composite for Simultaneous High-Efficiency PM and VOC Removal [84,85,86,87].

#### 2.1.8. Acrylonitrile Butadiene Styrene (ABS)

**Figure 10:**
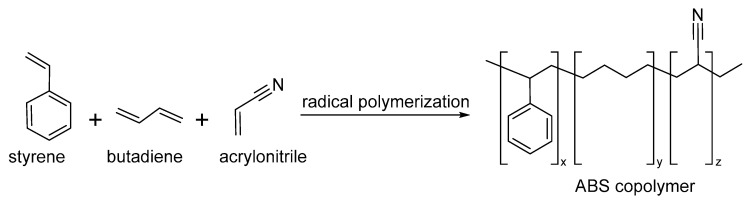
Synthesis of the ABS copolymer.

ABS is a thermoplastic copolymer consisting of three different monomer units, acrylonitrile (ACN), butadiene (BTD) and styrene (STE) (Figure 10) [88], where their proportions can vary from 15% to 35% CAN, 5% to 30% BTD and 40% to 60% STE. The monomers in the polymeric chain play a different role, the nitrile group provides strength, ACN contributes hardness, rigidity and heat deflection temperature, and butadiene provides toughness and ductility at low temperatures. In this sense, ABS combines the resilience of polybutadiene with the hardness and rigidity of polyacrylonitrile and polystyrene [89]. Two methods are used for the preparation of ABS copolymer, the mechanical blending of styrene-acrylonitrile resin (SAN) with a butadiene base elastomer butadiene/acrylonitrile rubber and grafting of styrene and acrylonitrile onto PB [90]. The preparation of ABS membranes is an interesting field of study due the permeate flux, rejection of the pollution indices and thermal resistance, flowability, and emissions of volatile organic compounds (VOCs) [91,92]. Moreover, electrospun ABS nanofiber films have been developed as a nanosorbent for head space thin film microextraction of HAPS with applications in samples of water or urine, and showed excellent extraction efficiency and nanofibers membranes for air filtration media [93,94].

#### 2.1.9. Polyurethane (PU)

**Figure 11:**
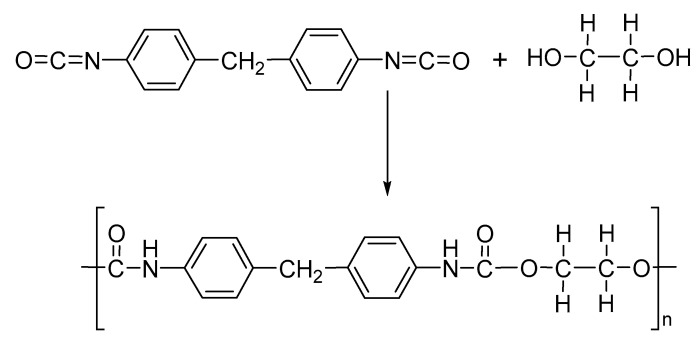
Synthesis of polyurethane.

PU are synthesized in a single step by reacting diisocyanates, polyols and catalyst as DABCO, metallic soaps or dibutyltin dilaurate (Figure 11) [95,96]. The main aliphatic isocyanates for the synthesis of PU are diphenylmethane diisocyanate (MDI) or toluene diisocyanate (TDI), hexamethylene diisocyanate (HDI) or isophorone diisocyantate (IPDI). However, the polyols can be polyether polyols and polyester polyols as ethylene glycol, propylene glycol, poly-ethylene propylene oxide, 1,4-butane diol and 1,6-hexane diol [97]. PU shows important types of applications and the development of versatile materials as thermoplastics, foams, powder coatings, paints, elastomers, and insulators [98,99,100]. However, the preparation of polyurethane nanofibers by electrospinning is a field of interesting study, due to vast possibilities for functionalization with high surface area to volume or mass ratio, ease of use, adaptability and potential applications in biomedical, filtration technologies, sensors and nanoweb lamination based on electrospun PU polymer nanofibers [97,101,102,103].

#### 2.1.10. Polyethylene Glycol (PEG)

**Figure 12:**
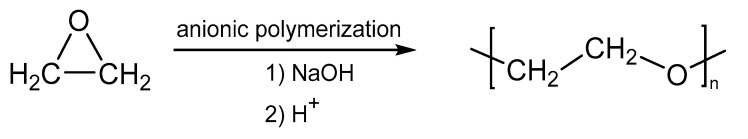
Synthesis of PEG.

PEG, also known as polyethylene oxide (PEO) or polyoxyethylene (POE), depending on its molecular weight, is an polyether polymer, initially produced by the reaction of ethylene oxide with water, ethylene glycol, or ethylene glycol oligomers (Figure 12) [104]. The reaction is a catalyst by alkalis or metal oxides that affect the low polydispersity and growing polymer chain in the polycondensation process [105]. Additionally, these polymers also are considered green organic solvents, and are promising solvents for sustainable organic synthesis [106] and have been used for the development of eco-friendly reactions [107]. PEG have also been used to prepare novel PEG derivatives as PEGylated−peptide biopolymer conjugates, hydrogels of PEG/DEPEG, PEG-tosylate, -mesylate, -bromide or aldehyde and HS–PEG–alkyne [105,108,109,110,111].

In combination with other polymers, they have been used for different applications, for example, electrospun nanofibers of polyamide-PEG have been used for headspace solid-phase microextration, blend with polycaprolactone (PCL) and poly(ethylene glycol) have been used to improve materials of human osteoblast maturation, hydrophilic–hydrophobic terpolymers containing PEG for tissue engineering and electrospun cellulose acetate butyrate/polyethylene glycol (CAB/PEG) composite nanofibers biodegradable that enhanced the cell adhesion [112,113,114,115,116].

#### 2.1.11. Polyethylene Terephthalate (PET)

**Figure 13:**
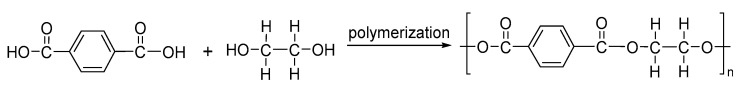
Synthesis of PET.

PET is a thermoplastic polymer widely used to make plastic bottles, polyester yarn, microfiber towels, cleaning cloths and a wide variety of plastic products. PET can be synthetized by the direct reaction of Fischer esterification between terephthalic acid and ethylene glycol or transesterification reaction where one ester is transformed into another by reacting dimeth ylterephthalate with ethylene glycol (Figure 13) [117]. A big problem with the production of PET is that it uses large amounts of derivatives of petroleum, making it an environmentally unfriendly polymer [118,119]. In response to the potential polymer pollution several applications have been proposed by chemical and mechanical recycling [120,121,122,123]. In this sense, recycling of PET has been studied for the development of aggregates such as the synthesis of copolyesters (PET/poly-Ɛ-caprolactone), synthesis of PET from biomass-based in ethylene glycol and glycolysis [124,125,126,127]. Different types of PET nanofibers have been developed with different applications, for example PET/SiO_2_ nanofibers membrane for applied to needle-felt filters, PET nanofibers loaded with silver nanoparticles for antimicrobial applications, PET nanofibers chemically modified with silane molecules for electroless deposition methods with copper and PET nanofibers as new adsorbent for micro-solid phase extraction of chromium(vi) in environmental water samples [128,129,130,131].

#### 2.1.12. Polyamide-6 (PA-6)

**Figure 14:**
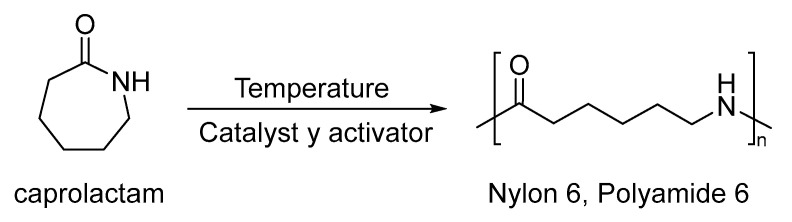
Synthesis of the anionic polymerization of PA-6.

PA-6 is a versatile thermoplastic polymer, very popular for the excellent mechanical properties such as impact strength, stiffness and high thermal degradation temperature, due the strong interchain attraction derived from the polarity of amide groups [132]. Among the different types of polyamides, nylon 6,6 is one of the most well known and commercialized aliphatic polyamides [133]. Several methods of polymerization have been used to synthetize PA-6, among them the transesterification by combination [134], direct polycondensation with triphenylphosphine melt-polymerization of block copolymers consisting of PA6 and Poly(N-cyclohexylmaleimide) (PCHMI) [132] and anionic ring-opening and condensation reactions (Figure 14) [135]. In the same way, different monomers can be used, such as poly(4,4′-diphenylsulfone terephthalamide) (PSA), poly(*p*-diphenyl oxide terephthalamide) (POA), poly(*p*-diphenylmethane terephthalamide) (PMA), and isophthaloyl chloride (IPC) to obtain nylon 6,6 copolymers [136]. Several applications have been developed taking into account PA-6 as an air filter for multi-level physical sieving of airborne particles via sequential electrospinning [134], as well as PA-6 nanofibrous nonwovens membranes, useful for separation systems with good mechanical properties, such as a high-tensile strength and elongation [137].

### 2.2. Electrospun Nanofibers Synthesis

#### 2.2.1. Nanofibers Synthesis by Electrosppining in Solution

The electrospinning process using polyacrylonitrile (PAN) has been the most studied for air purification and water decontamination applications. There are several parameters that must be studied including flow rate, distance needle-collector, voltage, etc. [138,139]. Although there are others polymers that will be mentioned below (See Table 1).

As an example of production conditions of PAN membranes there are: Polymer concentration at 8% dissolved in N,N-dimethylacetamide (DMAc), voltage at needle 55 kV and at the collector −5 kV, with a distance between them of 18.8 cm, under a flow rate of 15 mm min^−1^, at 23 °C. Although low air filtration efficiency was achieved, these membranes proved to be suitable for water filtration [140].

**Figure 15:**
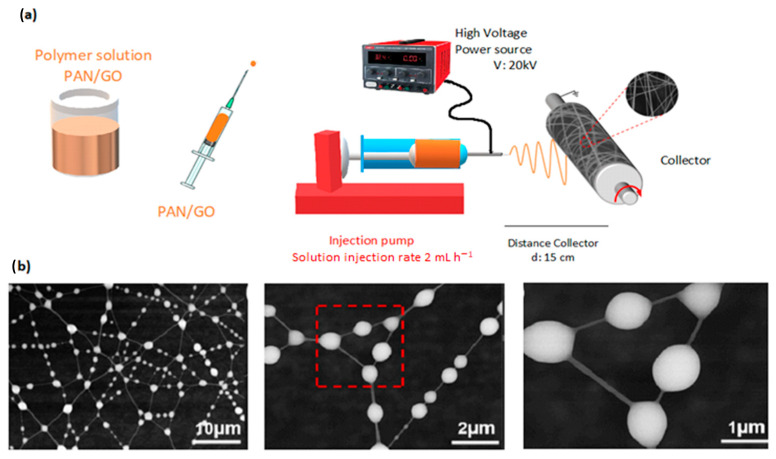
(**a**) Process for obtaining electrospun fiber (PAN/GO) in solution. (**b**) SEM images of the PAN/GO nanofibers after PM_2,5_ adsorption. Reprinted with permission from Ref. [144].

PAN can be modified with different additives to change a specific characteristic; for instance, graphene oxide (GO) allows for the trapping of the smaller particles [141,142]. In his study, N,N-Dimethylformamide (DMF) is used as a solvent and the electrospinning system is carried out with two needles, multilevel membranes were produced, with the application of a voltage of 15 kV, flow rate 0.8 mL h^−1^, needle-collector distance of 22 cm and 30 min of collection. A 99% efficient membrane was gotten in the air filtration and also, produced a low pressure drop [143]. Another study addressed the inclusion of polyimide to the mixture (PAN/GO) that was electrospun with a voltage of 20 kV, flow of 2 mL h^−1^ and distance of 15 cm. A filtration efficiency of 99.5%, pressure drop of 92 Pa and good thermal stability up to 300 °C were achieved. The electrospun process are shown in Figure 15 [144].

In order to increase the membrane porosity and to obtain a higher filtration efficiency with a lower pressure drop PAN has been modified with silicates. The distance between the collector drum and the needle was 18 cm, the feed rate was 0.3 mL h^−1^ and the applied voltage was 13 kV, at 25 °C [145].

Furthermore, the effect of the beads on the membranes has also been evaluated, for this 3% to 9% PAN in DMF was used, a membrane was built with polymer bilayer, a non-beaded layer and beaded layer, similar results were obtained to the single-layer membrane and commercial filters in terms of filtration efficiency, but a lower pressure drop was achieved [19]. However, in a different study, it was determined that a single-layer beaded membrane can reach efficiencies higher than 99% and low pressure drops of around 27 Pa [146].

It has been shown that, in the presence of ammonium tetrathiomolybdate (ATTM) in a PAN polymeric matrix when developing membranes, there is an improvement in the performance of air filtration and in the photocatalytic properties of the material, these allow us to capture toxic particles from the air and simultaneously offers self-cleaning properties when exposed to sunlight. A rotary drum was used and a voltage of 13 kV was applied, collection distance was 18 cm, with a flow rate of 0.3 mL h^−1^, and the operating temperature was 25 °C [147].

As mentioned above, it can be made of modifications to the technique, instead of modifying the polymer; with the aim of modifying the diameter and porosity of the fibers [148,149]. For example, it is possible to electrospin PAN without a needle with a double ring slit spinneret. The flow rate used was 20 mL h^−1^, voltage 70 kV and was collected in a rotary drum at 70 rpm. A filtration efficiency of 99% was achieved [150].

Another compound commonly used in electrospinning in solution is thermoplastic polyurethane (TPU) [151]. These polymer membranes were produced with a multi-needle electrospinning system, as this allows for an increase in the production efficiency of the membrane. 17% TPU was prepared in DMF and a voltage of 60 kV was applied and the reception distance was 30 cm. A filtration efficiency greater than 99.8% was achieved [152].

Unlike the aforementioned polymers, there are biodegradable polymers such as polyvinyl alcohol (PVA) that are used for the elaboration of eco-friendly membranes [153]. Electrospinning of 8% PVA has been tested at a flow rate of 0.2 mL h^−1^, a voltage of 10.02 kV, distance of 9 cm with a rotary collector at 150 rpm, at a controlled temperature of 27 °C. A pressure drop of 147 Pa was achieved for PM_10_ [154]. However, PVA is usually modified by the addition of a compound, or by structuring multilevel membranes with other membranes [155]. However, it is known that sodium lignosulfonate can preserve the ecological characteristics of PVA, and also improves the mechanical and filtration performance of the membrane. This additive has resulted in the production of efficient membranes for PM_2.5_ retention, under the effect of a voltage of 18 kV, distance was 15 cm, the flow rate was 0.6 mL h^−1^, and a rotary collector at 130 rpm [156]. Instead, to retain PM_1.0_, tannic acid is used as a PVA additive, which allows for the formation of much thinner fibers, the fibers were formed with the following parameters: Voltage of 15 kV, a distance of 15 cm, a flow rate of 0.5 mL h^−1^, and a rotary collector at 130 rpm [157].

Temperature is an important factor in the formation of membranes that is necessary to control, since the evaporation of the solvent affects the porosity of the fibers [21]. For filtration processes at high temperatures, thermally stable polymers are used, such as meta-aramid 1313 (MA1313), which must be mixed with LiCl in order to achieve efficient electrospinning. The parameters for the formation of this membrane are needle-collector distance, 15 cm, potential difference, 25 kV, propulsion speed, 50 μm s^−1^, temperature 25 °C, rotary collector speed, 40 rpm and collection time, and 3 h. In air filtration tests, it was determined that MA313 fibers maintained their mechanical properties during 129 h of exposure at 200 °C, in addition a minimum removal efficiency of 99% was achieved [158].

Polytetrafluoroethylene (PTFE) is also considered a temperature-resistant polymer; however, it is not a good material for spinning either, so it is used PVA and boric acid (BA) with a voltage of 15 kV, 15 cm of needle-collector distance, flow of 0.5 mL h^−1^, collection time of 8 h in a stainless steel mesh. 98% filtration efficiency and a pressure drop of 90 Pa [159]. On the opposite side Lin et al., 2019, studied the effect of low temperatures by electrospinning membranes from polyvinylidene fluoride (PVDF) dissolved at 6% in N-methylpyrrolidone (NMP) and acetone, a potential difference of 6 to 8 kV and a flow of 200 to 400 μL for 6 h were applied, the experiment was conducted at room temperature, however, the collector was cooled to −9 °C. It was determined that the porosity of the membrane can increase up to three times and that the pressure drop can decrease up to five times compared to membranes produced at 25 °C [160].

To improve filtration efficiency, membrane electrospinning with fiber diameter control has been tested, since it is directly related to filtration efficiency and depends on the intrinsic properties of each polymer [161]. However, it is difficult to control the diameter without adding other substances. Therefore, membranes have been produced in which the process variables were optimized through the experimental design. A membrane with a fiber diameter around 77 nm was obtained, which can retain PM_2.5_. 8% *w*/*w* of PAN solution, 12 kV voltage, rotary collector speed 5000 rpm, tip to collector distance 12 cm, flow rate 0.6 mL h^−1^ and electrospinning temperature 50 °C [5]. The PVDF has a high dielectric constant, which being under the effect of the electric field produces a great stretch and as a result very fine fibers are obtained, however it is not very easy to spin. Otherwise, PAN improves this property and allows for a better entanglement of the chains [14]. The research showed that, with 5% of PAN, it is possible to obtain a lower pressure drop, but to obtain a better filtering efficiency, it was necessary to add lithium chloride (LiCl) 0.7% to modify the surface load and decrease the diameter of the fiber, the experiment was carried out under the effect of 16 kV, with a flow rate of 0.03 mm min^−1^, the distance from the needle to the collector was 19 cm temperature of 30 °C and the speed of the rotary drum was 5 m min^−1^ [14].

Regarding the very small particles, PVDF membranes with the self-polarized ferroelectric phase can be produced, which have the advantage of retaining PM_0.3_, causing respiratory diseases, lung cancer and eventually death. The membrane was prepared with PVDF dissolved at 15% in DMAc and 2-butanone (MEK) in 5:5 ratio. To control the diameter size, sodium dodecyl sulfate (SDS) was used as a conductive surfactant. Electrospinning was performed with a flow rate of 0.5 mL h^−1^, voltage of 28 kV, and collecting needle distance of 16 cm [4]. Similarly, when using membranes with PVC and polyamide 6 (PA6) fibers, the triboelectric effect between the two nanofiber membranes occurs and generates electrostatic charges under the action of air vibration, thus improving electrostatic adsorption with pressure drop [162].

Despite the advantages mentioned about electrospinning, in some cases there are fragile membranes due to non-woven fibers. Therefore, it has been proposed to use epoxy resin (4,40-diaminodiphenyl sulfone) that allows to reinforce polyimide membranes (based on polyamic acid, PAA), to achieve the objective the PAA membrane was immersed in the resin and the cross-linking occurred that gave it a resistance four times greater than the PAA membrane [163]. Something similar happens with polyethersulfone (PES), which, to improve its ability to form membranes, the modification of its properties was studied by adding polyamide 66 (PA66). PA66 was prepared at 18% in formic acid, and PES at 25% in DMF, the two solutions were electrospun simultaneously on the same collector, with PES with a flow of 0.5 mL h^−1^ was fixed, the voltage was 16 kV and the distance from the needle to the rotary collector was 18 cm. While for PA66 a flow of 0.6 mL h^−1^ was fixed, a voltage of 16 kV and the distance was 20 cm. This membrane achieved a PM_0.3_ retention of 99.999% [164].

Filters can acquire antimicrobial properties, for example, 12% PA6 in acetic acid/formic acid in 2:1 ratio is modified with 1 dodecyltrimethylammonium bromide (DTAB) and electrospinning was performed at 25 °C, with a voltage in the needle of 60 kV and −30 kV in the collector and a distance between them of 24 cm, at a flow rate it was 30 mm min^−1^. After an air blowing test, it was determined that the DTAB imparts its antimicrobial properties to the membrane [165].

Additionally, it has been proven that the inclusion of β-cyclodextrin (β-CD) in polymer membranes favors the retention of volatile organic compounds VOCs [87,139,166]. For this reason, the use of PVA modified with a menthol/β-CD complex has been tested, with possible application in tobacco filters, since these are very dangerous for health. Polymer solutions were made from 8% PVA in deionized water and 0.4% sodium dodecyl benzene sulfonate (SDBS) was added; then, it was added to 5%, 10%, 15% and 20% of the menthol/β-CD inclusion complex. These were electrospun with a voltage of 16 kV, distance of 15 cm, flow rate of 0.3 mL h^−1^, collection time on aluminum foil was 5 h, the experiments were carried out at 20 °C. The resulting fibers were coupled to a cigarette filter and nicotine and tar was retained, however CO could not be retained [167].

The electrospinning technique has also been used to improve conventional filters, as in the case of masks made of polypropylene, these usually wear out and over time the filtration efficiency decreases. To increase the efficiency of these inputs, PVDF was electrospinned at 13, 14 and 15% *w*/*w* in N,N-DMF onto a non-woven polypropylene layer, electrified and blown by fusion, the variables used were: Voltage of 27, 28 and 29 kV; needle-collector distance 16, 19 and 22 cm. By adding the PVDF layer, it was possible to increase the filtration efficiency from 70% to 95% [24]. Additionally, in response to the emergence of COVID-19, alternatives to conventional masks that are friendly to the environment have been developed. Cellulose acetate (CA) is a biodegradable and reusable material because it is considered a good candidate to produce filters by electrospinning [168], however it needs to be improved with LiCl and TPU [169]. An acetone/DMAc 3:1 mixture was used as a solvent. CA at 14% and TPU at 18% were prepared and mixed in a 1:1 ratio; 1% LiCl was added. The electrospinning was carried out with a flow rate of 1 mL h^−1^, needle-collector distance of 18 cm, voltage of 20 kV, speed of the rotary collector of 200 rpm, at a temperature of 27 °C. The membrane achieved a filtration efficiency of 99.8%, and a pressure drop of 52 Pa. Additionally, it was stable and reusable [170].

Related to natural fibers, studies with silk fibroin are reported, membranes based on this material were produced with a concentration of 8% fibroin, PEO was used as a precursor of electrospinning in 1:4 ratio (silk/PEO), voltages of 10 to 20 kV were applied, and the other parameters were adjusted depending on the type of fluid. A filtration efficiency of 99.98% and a pressure drop of 75 Pa were achieved [171]. Chitosan is a biocompatible compound whose fibers are very fragile, for this reason PVA is added. For the elaboration of membranes based on this compound, 2% chitosan is mixed (in acetic acid 1%) with PVA 13% (in water) in relation 0/100, 10/90, 20/80, and 30/70. Membranes are obtained with fibers of low diameter and high porosity [172].

Given the problem of waste management, once the filters are discarded, the possibility of producing biodegradable membranes has been investigated [171,173,174]. Shellac is a polymer used as a low-cost but fragile food additive, however when mixed with polyvinylpyrrolidone (PVP) filtration efficiencies of 95% and 98.1% are achieved for PM_2.5_ and PM_10_ respectively, that is, 30% and 40% more efficient than a commercial surgical mask, to achieve this result, 40% shellac and 8% RRP were prepared, both in ethanol. They were mixed in 3:1 ratio (shellac: PVP). The electrospinning was carried out with voltage of 15 kV, flow of 1 mL h^−1^, needle-collector distance was 13 cm, and the polymer was collected in a 150 stainless steel mesh at a temperature of 25 °C [175].

From another point of view, the recycling of waste, particularly expanded polystyrene waste from food packaging, has been raised [176,177]. The clean polymer was dissolved in the d-limonene/DMF mixture (1:1 ratio) in concentrations of 15%, 20% and 25%. The electrospinning was carried out with a flow rate of 13 μL min^−1^, voltage of 15 kV, 10 cm needle-collector distance, and a rotary collector provided with a 150 stainless steel mesh. In general, it was determined that polystyrene does not degrade during electrospinning and that the fibers obtained reach filtration efficiencies greater than 95% and very low pressure drops, around 0.15 Pa [178].

In order to recycle wool waste, keratin membranes have been developed with polyethylene oxide (PEO) as an additive, because keratin protein is difficult to extract and has low structural stability, therefore electrospinning is difficult. PEO gives it the stability needed to produce membranes. To obtain keratin, wool was dissolved in a solution with sodium sulfate, urea and SDS; the protein was extracted and mixed with PEO; a membrane was then produced using the following parameters: Voltage,14 kV, receiving distance, 24 cm and rotational flow, 0.2 mL h^−1^; and it achieved a filtration efficiency of 88% [179].

Metal-organic framework (MOFs) have also been included in the modification of polymers for electrospinning, due to their potential applications in gas separation; particularly the zeolitic imidazolate framework-8 (ZIF-8) is used to modify polyacrylic acid [180], polyimide (pi) [181] and PAN [182]. In all three cases, it was applied to retain PM_2.5_ with filtration efficiencies of 99.6, 96.6 and 99.9, respectively.

Finally, membranes of ceramic materials can be produced by electrospinning, with the help of a binding polymer, which is removed by calcination, and it also produces a change in the structure of the ceramic material, from amorphous to polycrystalline. The main advantage of this type of membrane is the resistance and duration provided by the material. Zirconium propoxide (ZrP), yttrium nitrate hexahydrate (Y), tetraethyl orthosilicate (TEOS) and PVP were used. The electrospinning parameters were temperature 25 °C, flow rate 1 mL h^−1^, voltage of 20 kV, and distance 10 cm. It was shown that the diameter of ceramic membranes can be reduced, by decreasing the amount of binder. Membranes that can replace conventional water and air filters were produced [183].

Table 1 shows a summary of electrospinning synthesis conditions, and it is possible to find the polymer preparation procedure, parameters of synthesis and the main result in each study.

**Table 1:** Additional research concerning electrospinning for air filtration.

Polymer Conditions	Electrospinning Parameters	Result	Ref.
8% PAN dissolved in DMF and 11% PMIA (m-phenylene isophthalamide) dissolved in LiCl (2% LiCl into DMAc)	Voltage: 55 kV Distance 18 cm Collector: rotating metal roller, at 70 rpm	The membranes, even after being exposed to high temperatures (140 to 220 °C) keep diameters around 100 nm and reach filtration efficiencies of 99%.	[184]
14% PAN	Inner diameter needle: 0.5 mm Feed rate: 0.4 mL h^−1^ Voltage: 13 kV Distance: 20 cm Collector: aluminum foil, embossed paper, and a bulged bubble. Temperature: 25 °C	The collector influences the distribution of the electric field and nanofibers, the fibers collected in embossed paper resulted in greater filtration efficiency and with smaller pore size, and those collected in bulging bubbles resulted in a smaller pore size.	[185]
30% β–CD into a polymer solution (8% PAN in DMF).	Collector: rotating metal roller 13 cm × 19 cm, at 30 mm s^−1^. Distance 20 cm	The membranes were efficient in the adsorption of VOCs, formaldehyde and xylene. Achieved a filtration efficiency of over 95% and a low pressure drop (112 Pa)	[139]
6% PAN in DMF 15% PA6 in formic acid	Multistructured composition PA6/PAN/PA6 Distance 20 cm Collector: rotating metal roller at 50 rpm Voltage 30 kV Feed rate PA6: 0.3 mL h^−1^ Feed rate PAN: 1 mL h^−1^	A multistructured membrane with integrated properties of the ultrafine diameter of PA6 and PAN beads was obtained, filtration efficiencies of 99.99% and pressure drops of 117.5 Pa were achieved for nanoparticles from 300 to 500 nm.	[186]
15% β–CD in the polymer solution (8% PAN in DMF).	Voltage 18 kV Feed rate 0.5 mL h^−1^ Distance 20 cm Collector: rotating metal roller at 180 rpm Collection time: 120 min	It was achieved: Fiber diameter between 305 to 463 nm. Benzene filtration efficiency greater than 95%. Pressure drop from 92 to 164 Pa.	[166]
Uni1: 10% PAN/PVDF with ratio 6:4 Uni2:12% PAN/PVDF with ratio 6:4 Bi: 12% PAN/PVDF with ratio 8:2 and 10% PAN/PVDF with ratio 6:4.	Multineedled electrospinning Configuration: Uni1/Bi/Uni2 Voltage 60 kV Rate: 6 m min^−1^	The filter with sandwich structure showed an excellent filtration efficiency of 99.984% for PM 0.26 with, a pressure drop of 85.02 Pa.	[187]
PVDF was prepared in DMF with 0, 0.5 and 1% of DTAB	Voltage: 60 kV Distance: 18 cm Collector: rotating metal roller at 60 mm min^−1^ Temperature: 22.5 °C	PVDF modification allowed a homogenous membrane production. The 1% DTBA membrane achieved a complete inhibition against Staphylococcus aureus subsp. Aureus	[188]
12, 15 and 18% PVC were prepared in DMF/tetrahydrofuran (THF) in ratio 1:1. 6% PAN was dissolved in DMF	Bead-on-string structure Voltage: from 9 to 10.5 kV Collector: rotating metal roller at 50 rpm Distance 20 cm Collection time: from 0.05 to 0.15 mm min^−1^	An ideal hydrophobic filter was obtained to retain particulate matter in environments with high relative humidity, since the pressure drop is reduced, compared to hydrophilic filters.	[189]
21%, 24%, 27%, and 30% of Poly (arylene sulfide sulfone) (PASS) was prepared in NMP	Voltage: 15 kV Distance: 20 cm Flow rate 0.5 mL h^−1^	The PASS membrane had a diameter of 0.31 μm and a base weight of 3 g/m^2^. With a high particle removal efficiency (99.98%), low pressure drop (68 Pa).	[190]
5 mg of Magnesium Tetraphenylporphyrin (MgTPP) was added to 12% of Polyetherimide (PEI) solution (dissolved in DMF/THF, in ratio 1:1)	Voltage: 17.2 kV Flow rate: 1.5 mL h^−1^ Distance: 10 cm	The diameter of the nanofibers was in the range of 1.2 to 1.4 μm and had a thermal stability up to 411 °C. In addition, a CO2 and PM_2.5_ filtration capacity of 74% and 81% respectively was achieved.	[191]
5, 10, 15, 20, 25, and 30% of polystyrene (PS) in DMF PA-6 solutions at concentrations of 10, 15, 20 and 25% of PA-6 were prepared in FA 5, 10, 15, and 20% (TPU) were dissolved in DMF/THF (1/1, *v*/*v*)	Collector: rotating metal roller covered with a polypropylene piece, at 180 rpm. Distance 15 cm Rate: 100 mm cm^−1^ Temperature 25 °C	A submicron fibrous layer of TPU, a pearly fibrous layer of PS and an ultrafine nanofibrous layer of PA-6 were successively prepared. A filtration efficiency of up to 99.99% and a low pressure drop of 54 Pa were achieved. After deterioration, a filtration efficiency of more than 70% was achieved.	[192]
4.85 mg of GO was dispersed in 2 mL of DMF. Then, 2 mL of acetone was added. Subsequently, 0.97 g of PVDF powder was slowly added to the GO dispersion	Voltage 8.5 kV Injection rate 1 mL h^−1^ Collector: rotating roller at 60 rpm Distance 15 cm Temperature 25 °C	GO/PVDF membranes show a higher PM_2.5_ removal efficiency (99.31%) and better reusability than the pristine PVDF NFMs (93.74%).	[193]
Poly(lactic acid) PLA was prepared at 8% in dichloromethane (DCM)/DMF ratio 80:20. methyl-(β-CD) was added	Voltage 16 kV Distance 10 cm Flow rate 0.5 mL h^−1^	A filtration efficiency greater than 98% and pressure drop of 30 Pa were achieved for Toluene (a VOC).	[87]
0.5% of Tetrabutylammonium chloride (TBAC) was dissolved in Dimethyl carbonate (DMC)/DMF (7/3), then PLA was added at concentration of 3%	Voltage: 25 kV Distance: 18 cm Feed: 5 mm min^−1^ Collector: rotating roller at 0.04 to 0.2 m min^−1^	A mask with filtration efficiency for PM0.3 of 99.996% and a low pressure drop (104 Pa) higher than the commercial filter N95 was developed. Additionally, it has natural biodegradability based on PLA	[194]
8, 10, 12 and 14% PLA in DMF	Collector: aluminum foil or Recycled Poly(ethylene terephthalate) (R-PET) webs.	A filtration efficiency of 99.992% was achieved, a low pressure drop of 201.11 Pa.	[195]
Zeolitic imidazolate framework crystals (ZIF) (0.125 g) were dispersed in 2 g of DMF by ultrasonic treatment, and then 0.4 g of soluble PU was added.	Distance 15 cm Voltage 18 kV Feed rate 0.6 mL h^−1^ Temperature 25 °C	Filtration efficiencies greater than 99% were achieved for PM2.5 and pressure drop of 400 Pa.	[196]
12% of Polyamide 66 (PA 66) in formic acid	Voltage: 16 kV Distance: 20 cm Feed: 0.5 mm h^−1^ Collector: copper mesh	200 nm diameter nanofibers were obtained. Additionally, it was achieved a filtration efficiency of 99.99% for 0.3 µm particles.	[197]
19% of PEO in a mixture water/ethanol (1/1) 9% of PVA in water	Voltage: 8 kV Distance: 8 cm Feed: 100 µL h^−1^ Voltage: 15 kV Distance: 15 cm Feed: 200 µL h^−1^	PVA membrane had a better membrane efficiency than PEO, 97.6% and 92.8%, respectively.	[198]
8% of PVA in water	Voltage: 10.02 kV Distance: 9 cm Feed: 200 µL h^−1^ Temperature: 27 °C Collector: rotating roller at 15 rev min^−1^	PVA nanofibrous membrane had the highest filtration efficiency (99.9%) for the particles under 10 µm compared with two conventional filter (23.6 and 99.1%)	[154]
14% of PVDF mixed with 0, 0.5, 1 and 2% of lead zirconate titanate (PZT). The mixture was dissolved in DMF/acetone (1/1).	Voltage: 20 kV Distance: 10 cm Feed: 1 mL h^−1^	An efficiency of 98.51% for particles from 50–500 nm.	[199]

#### 2.2.2. Nanofibers Synthesis by Melt Electrosppining

The melt electrospinning process is known as solvent-free electrospinning. This process has reduced the use of toxic solvents and it is considered a green technology. The membrane is obtained by directly heating the polymers, where uniform membranes with good tensile strength, small diameter and free of solvents can be obtained [200,201,202]. There are some polymers that have been used by melt electrospinning that will be mentioned below (See Table 2).

An example of electrospinning by melt are membranes of polylactic acid (PLA), which were obtained with the addition of 6% acetyl tributyl citrate non-toxic (ATBC) by passing a flow of hot air of 25 m s^−1^ at 240 °C. Membranes with a diameter of 236 nm of excellent stability and without thermal degradation were achieved [203]. Voltage: 40–45 kV.

**Figure 16:**
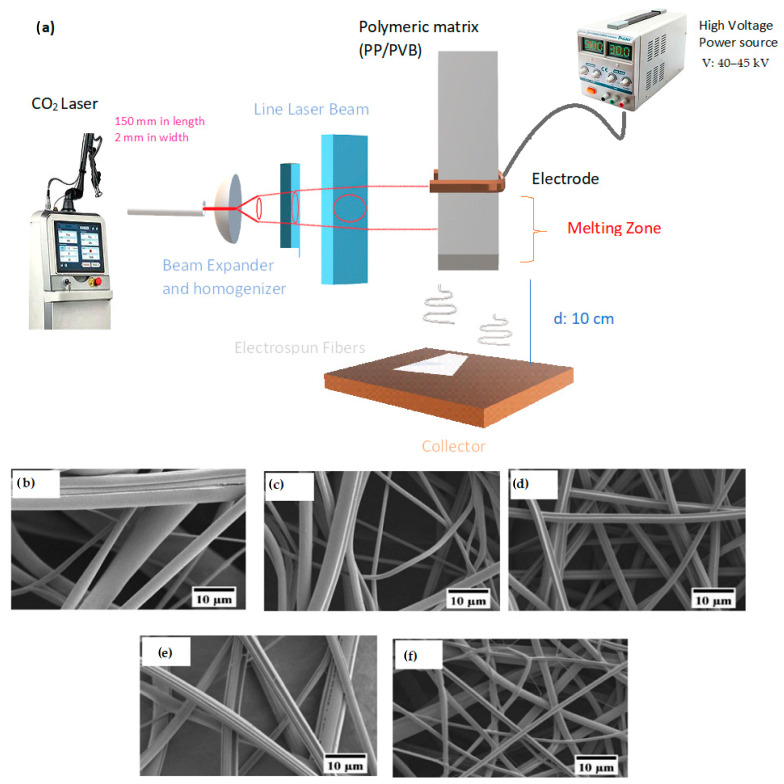
(**a**) CO_2_ Laser system for obtained fiber PP/PVB by Melt Electrospinning. SEM images of PP fibers from: (**b**) PVB-10%; (**c**) PVB-20%; (**d**) PVB-30%; (**e**) PVB-40%; and (**f**) PVB-50%. Reprinted with permission from Ref. [204].

Polypropylene (PP) is considered a temperature-resistant polymer; thus, it is used with polyvinyl butyral (PVB) to obtain melt electrospinning without a nozzle with the help of a CO_2_ laser-beam system, as shown in Figure 16. The resulting fibres with a diameter low of 181 ± 105 nm were obtained [204].

Additionally, poly(ether-block-amide) (PEBA) membranes with a diameter of 1.92 ± 3.31 nm have been obtained. The production conditions of PEBA membranes are: Collector distance of 6 cm, voltage 20 kV, collector speed 55 rpm, and melting temperature of 270 °C. The results obtained showed that the operating parameters, such as voltage, distance, collector speed and melting temperature influenced the fibre diameter. The mentioned values were the limit in which the fibre diameter decreased, but above these parameters, the diameter of the electrospun membranes increased. Due to its properties, the membranes can be applied for hydrophobic packing, separation, evaporation, and osmotic membranes [202].

The melt electrospinning technique has been used to achieve air filters, as in the case of Polymide 12 (Evonik Industries AG, Essen, Germany, Vestamid™ L 1600). These were electrospun by the combined melt-solution method were obtained through a prototype equipment. On the melt electrospinning side, the polymer Polymide 12 passed through a collection system, where conditions were voltage of 25 kV, a flow rate of 0.6 g h^−1^, collector distance of 7 cm, and the experiments were carried out at 300 °C. While the electrospinning in solution was carried out through a syringe-pump with Polymide (PA)6/6 at voltage of 25 kV, flow rate of 0.2 mL h^−1^ and collector distance of 10 cm. In the process, nanofibers with a porosity of 94.78% were obtained. The filtration quality factor between 0.068–0.085 Pa^−1^ for PM_1_ particles with low values of pressure drop (15.92–50.17 Pa) and efficiency of 93.7%. PM_1_ and 98.5% PM_10_ [205]. Additionally, membranes have been obtained through melt electrospinning combining polypropylene (PP) with polyvinyl alcohol (PVA) and 2.5% Zeolite imidazole Frameworks-8 (ZIF-8) with a filtration efficiency of 96.5% and factor of quality of 0.099 Pa^−1^ for PM_2.5_, with good tensile strength and low pressure drop [200]. Table 2 shows a summary of melt electrospinning synthesis conditions for the development of electrospun nanofiber membranes.

**Table 2:** Melt electrospinning for the development of nanofiber membranes.

Polymer Conditions	Electrospinning Parameters	Result	Ref.
PLA (polylactic acid) addition 6% ATBC (acetyl tributil citrate non-toxic)	Voltage: 40 kV Distance: 9 cm Airflow: 25 m s^−1^ Temperature of spinneret 240 °C	The membranes were stable, keep diameters around 236 nm. There was not thermal degradation of PLA	[203]
10% PP (polypropylene) 90% polyvinyl butyral (PVB)	CO_2_ Laser-beam Laser beam distribution: 150 mm in length 2 mm in width Feed rate: 1.0 mm min^−1^ Voltage: 40–45 kV Collector distance: 10 cm	Fiber diameter depended on the amount of PVB in blends. The diameter of fibers decreased with increased PVB content. Diameter of fibers low of 181 ± 105 nm	[204]
Poly(ether-block-amide) (PEBA) in glacial acetic acid or butanal	Distance 6 cm Collector: rotating metal roller at 55 rpm Voltage 20 kV Feed rate: 3 mL h^−1^ Melt temperature 270 °C	Melt electrospinning produced large fibres. keep diameters around 1.92 ± 3.31 nm	[202]
Polymide 12 (Melt electrospinning) 15% Polymide 6/6-85% formic acid	Melt process Voltage 25 kV Feed rate 0.6 g h^−1^ Distance 7 cm Melt temperature 300 °C Electrospinning in solution Voltage 25 kV Feed rate 0.2 mL h^−1^ Distance 10 cm	It was achieved: membranes with porosity of 94.78%, filtration efficiency greater than 93.7% PM_1_ and 98.5% PM_10_ with, a pressure drops of 15.91–50.17 Pa.	[205]
Polypropylene (PP) 10 % polyvinyl alcohol (PVA) 2.5% Zeolite imidazole Frameworks-8 (ZIF-8)	Hot Air temperature 210 °C Voltage 24 kV Feed rate 0.8 mL h^−1^ Distance 20 cm	The membrane showed good air permeability, high mechanical properties, and optimal PM_2,5_ filtration performance. It was achieved: filtration efficiency of 96.5% with factor of quality of 0.099 Pa^−1^, and pressure drop of 34 Pa.	[200]

### 2.3. Applications

Environmental pollution will head the list of top global issues facing society for the next 50 years and nanotechnology is responding to these challenges by designing and fabricating functional nanofibers optimized for energy and environmental applications. One of the main methods to prepare these nanofibers is based on electrospinning: A highly versatile method that allows for the fabrication of continuous fibers with diameters down to a few nanometers. This methodology has been applied to both natural and synthetic polymers, ceramics, and carbon [206]. Electrospinning is fundamentally different from air or other mechanically driven spinning techniques in that the extrusion force is generated by the interaction between the charged polymer fluid and an external applied electric field [102].

A method very similar to Electrospinnig is Electrostatic-Assisted Melt Blown, which is one of the commercial nonwoven technologies that allow, in one single step, to obtain fine fibers of the polymer (1–8 um). In this process, the polymer is fed into an extruder, and the molten material is propelled towards the filter before reaching the spinning head where the melted polymer is transformed into filaments by high-speed hot air and forms nonwovens fibers on the webformer [207].The use of synthetic polymers is widespread to reduce the environment pollution, and some papers report the preparation of polymeric nanofibers by electrospun, while other researchers report the synthesis by electrospun of the composite nanofiber membranes. Table 3 shows a list of nude and modified electrospun nanofibers used for the development of electrospun nanofiber membranes.

## 3. Conclusions

The electrospinning process is a promising method to fabricate air filters, with high filtration efficiency and low-pressure drop. Two important aspects can tribute or modify the membrane properties. First, the polymer and its composition can control thermal stability, and mechanical resistance, among others, this aspect includes the option to mix polymers or tack on additives to change or improve the membranes properties. Second, electrospinning parameters, such as, the distance between collector and needle, voltage, flow rate, temperature, and collector type are able to regulate diameter size and membrane porosity. However, it is possible to alter the technique in multineedle or needleless electrospinning. In addition, membrane configuration can modify and produce multistructured membranes.

## Figures and Tables

**Table 3:** A short list of nude and modified electrospun nanofibers, application to air purification.

Polymer	Product	Technique of Development of Nanofibers	Uses	Ref.
Polymethyl methacrylate (PMMA)	Electrospun nanofiber of PMMA/cyclodextrins	Electrospinning	Capture of VOCs	[41]
Polyvinylpyrrolidone (PVP)	Dual-size PVP−cellulose composite nanofibers	Via one-step electrospinning	multilayer air filters to capture aerosol particles	[208]
Polyacrylenitrile (PAN)	Electrospun nanofibers web of PAN	Needle-free wire electrospinning industrial production method.	Point-of-use water and air cleaning removing PM_2.5_ and PM_10_	[140]
Polystyrene (PS)	Polystyrene-SiO_2_ nanoparticle (PS-SNP) fibrous membrane	Electrospun PS fibers and SiO_2_ nanoparticles	Composite multi-layered filter masks with high air filtration and permeability.	[209]
Polyvinyl alcohol (PVA)	Electrospining nanofibres of Quaternary ammonium chitosan/PVA	Electrospun of composite nanofiber membranes.	Air purification and antimicrobial material.	[210]
Polypropylene (PP)	Polypropylene Micro and Nanofibers	Electrostatic-assisted melt-blown system	Filtration efficiency may be used in air filtration.	[207]
Polylactic acid (PLA)	Electrospun PLA-Cyclodextrins Composite	Electrospun of composite.	Removal of PM and VOC	[87]
Acrylonitrile butadiene styrene (ABS)	Nanofiber membranes from ABS	Electrospinning method	Air filter applications for remove PM_2.5_	[94]
Polyurethane (PU)	Transparent Polyurethane Nanofiber	rotating bead spinneret for large-scale electrospinning	Capture of fine particle matter.	[211]
Polyethylene glycol (PEG)	Electrospun PA/PEG nanofibers	Electrospinning	Headspace solid-phase microextraction	[112]
Polyethylene terephthalate (PET)	Electrospun microfiber membranes from PET	Electrospinning	Removal of viable aerosol nanoparticles like bacteria, fungi, and also viruses, mainly SARS-CoV-2	[212]
Polyamide-6 (PA-6)	Electrospun nanofiber from polysulfone/polyacrylonitrile/polyamide-6	Sequential electrospinning.	Filtration and separation	[213]

## Data Availability

The study did not report any data.
